# Fluctuating portal velocity tracing with rhythmicity: ultrasonic differential diagnosis and clinical significance

**DOI:** 10.2478/v10019-012-0028-9

**Published:** 2012-04-19

**Authors:** Qingxin Meng, Lei Lv, Bin Yang, Ninghua Fu, Guangming Lu

**Affiliations:** 1 Department of Ultrasound; 2 Department of Cardiology; 3 Department of Medical Imaging, Jinling Hospital, School of Medicine, Nanjing University, Nanjing, Jiangsu Province, China

**Keywords:** velocity tracing, portal hypertension, tricuspid regurgitation, arterioportal fistula, cardiogenic trans-sinusoidal shunting

## Abstract

**Background:**

To evaluate the usefulness of the routine sonographic evaluation of the pattern of fluctuate portal velocity tracings and the hepatic veins for the diagnosis of arterioportal fistula (APF) and cardiogenic trans-sinusoidal shunting (CTS).

**Materials and methods.:**

Color Doppler flow imaging and pulsed-wave Doppler (PW) examinations of the portal vein were performed in 282 subjects. The waveforms of the velocity tracings in the portal main trunk and its branches were determined to infer APF or CTS. Suspected cases of APFs or CTSs were always confirmed by echocardiography, contrast-enhanced ultrasound, computed tomography, or digital subtraction angiography findings. The portal maximum velocity (*V*_max_), minimum velocity(*V*_min_), *V*_max_/*V*_min_, arterial peak systolic velocity and resistance index, and venous reverse and forward velocities were used to estimate their haemodynamics.

**Results:**

The waveform of the velocity tracing for the draining portal vein of APF was typically arterial-like or diphase, as indicated by a systolic hepatofugal dwarf peak and a diastolic hepatopetal low flat shape. The flow in the affected portal vein was always hepatofugal in an intrahepatic patient, whereas a hepatopetal flow was observed in an extrahepatic APF patient. The waveform of the velocity tracing for the portal vein of CTS patients, especially its intrahepatic branches, showed a typical hump-like shape with or without a transitory hepatofugal tracing. The PW results displayed an increase in the retrograde phase of the hepatic venous flow with increased velocities in the two phases.

**Conclusions:**

Portal velocity tracings should be evaluated during routine detecting for APF or CTS, especially in patients with gastrointestinal upsets.

## Introduction

Arterioportal fistula (APF) and cardiogenic trans-sinusoidal shunting (CTS) may both lead to portal hypertension and subsequent esophageal varices with bleeding.^1^–^6^ A timely diagnosis is essential because most cases have curable causes of portal hypertension that require different therapeutic tools.^7^,^8^ However, APF and CTS are often ignored because some patients are asymptomatic or experience equivocal symptoms such as diarrhea, edema, ascites, abdominal pain, and distension. Some APFs exist in patients with liver cirrhosis, whose portal hypertension can be considered the common evolution of cirrhosis; and other patients have the normal internal diameters of portal vein, hepatic vein, and hepatic artery, with indiscoverable focus.^9^–^12^

APF and CTS can cause variations in hepatic haemodynamics, especially of the portal vein.^13^ Ultrasound (US) analysis, which is dynamic, noninvasive, economical, and convenient, can examine blood vessels in every direction.^14^ The haemodynamics determined from the velocity tracings detected via US can easily be analysed^15^, but a detecting diagnostic procedure that can be applied during routine examinations has yet to be properly defined. The present study was performed to determine the usefulness of the routine evaluation of portal velocity tracings, in combination with those of the hepatic artery and vein, for APF and CTS diagnosis.

## Materials and methods

### Patients and controls

Color Doppler flow imaging (CDFI) and pulsed-wave Doppler (PW) studies of the portal vein were performed on 195 patients with APF or CTS. These 195 patients were selected from a consecutive series of APF or CTS diagnoses between January 2002 and December 2010. Among 195 patients, 103 were males and 92 were females. The age distribution ranged from 26 to 67 years, with a mean age of 53 years. The clinical symptoms were the following: edema in 105 patients, abdominal pain and distension in 53 patients, ascites in 32 patients, diarrhea in 17 patients, and malignant hypertension in 3 patients ( diastolic pressure>140mmHg). 23 patients were asymptomatic. Among 87 control subjects, 41 were males and 46 were females. The age distribution ranged from 24 to 72 years, with a mean age of 49 years. No significant difference in sex or age distribution between the 195 patients and 87 control subjects.

### Standard pre-settings

Two-dimensional images with homogeneous gain could clearly display the vascular lumens. In the CDFI, the blood flow signals exactly suffused the vascular lumen with soft color. The sampling frame steer was consistent with the direction of blood stream, and the sampling gate located at the center of lumen, of which the width was 2 mm, and the correction line paralleled with the direction of bloodstream.

### CDFI and PW

**FIGURE 1:**
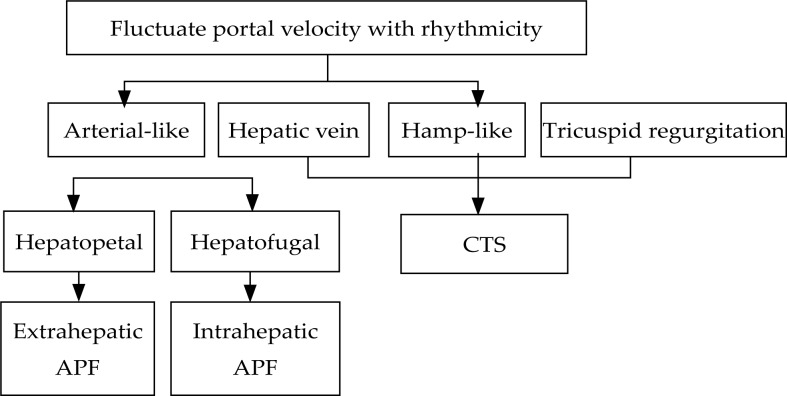
Diagnostic procedure for APF or CTS.

All subjects were evaluated by a skilled technician in the morning following an overnight fasting. The patients and control subjects routinely underwent CDFI and PW. The portal maximum velocity (*V*_max_), minimum velocity (*V*_min_) and *V*_max_*/V*_min_, arterial peak systolic velocity (*PSV*) and resistance index (*RI*), [(*V*_max_*-V*_min_)/*V*_max_], venous reverse velocity (*RV*) and forward velocity (*FV*), and reverse time/forward time [(*RT*)*/*(*FT*)] were used to estimate their haemodynamics. Each result is the mean of three measurements. The waveforms of the velocity tracings in the main trunk and the branches of the portal vein were determined to infer APF or CTS. A detecting diagnostic procedure is shown in Figure 1.

### Other procedures for confirmed diagnosis

Suspected APF or CTS based on color Doppler US findings were always confirmed by echocardiography, contrast-enhanced US (CEUS), computed tomography (CT), or digital subtraction angiography (DSA) findings.

CEUS was performed using an Acuson Sequoia 512 US scanner equipped with a 3–5 MHz convex transducer and CPS, a contrast-specific CEUS software (Siemens Medical Solutions, Mountain View, CA, USA). Standard pre-settings were used, with adjustment options for individual patients. After the baseline evaluation, SonoVue (Bracco, Milan, Italy) was injected intravenously as a bolus of 2.4 mL, followed by a flush of 5 mL of normal saline. The region of interest set in each JPEG file for the affected portal vein was observed in real-time for about 2 min after the intravenous injection of SonoVue. The entire process was recorded and saved on the hard disk attached to the scanner.

CT scans were performed with a dual-source CT system (Somatom Definition, Siemens Healthcare). Contrast-enhanced triple-phase scans (arterial, portal venous, and equilibrium phases) were obtained after an intravenous bolus injection of 120– 150 mL of iopromide (Ultravist 300 mg I/mL, Bayer Schering Pharma) at a rate of 4.0 mL/s using an 18-gauge catheter.

DSA was performed with femoral catheterisation by the Seldinger technique using a biplane DSA unit with rotational capabilities (Axiom Artis dTA, Siemens Healthcare). Typically, 6–9 mL of nonionic contrast medium (iopromide, Ultravist 300 mg I/mL) was used per acquisition. A baseline DSA of the coeliac trunk and the superior mesenteric artery was performed to visualize the liver vasculature and evaluate the portal vein patency. The common hepatic artery and the right and left hepatic arteries were cannulated, and more superselective cannulations of the feeders to the arteriovenous shunts were performed, if indicated, with or without the use of microcatheters. The arterial and early and late parenchymal phases were evaluated after the acquisition of digitally subtracted images at a rate of one per second. APF was considered in cases where an opacification of one or more portal branches before or during the early parenchymal phase was observed.

### Statistical analysis

All values are reported as mean ± standard deviation. Mean comparisons were performed using the t-test for paired samples, as appropriate. Comparisons of coefficients of variation were performed by F-tests. A value of *P* < 0.05 was used as the threshold for the statistical significance. A statistical analysis was performed using SPSS 16.0 software.

## Results

### US findings

**FIGURE 2:**
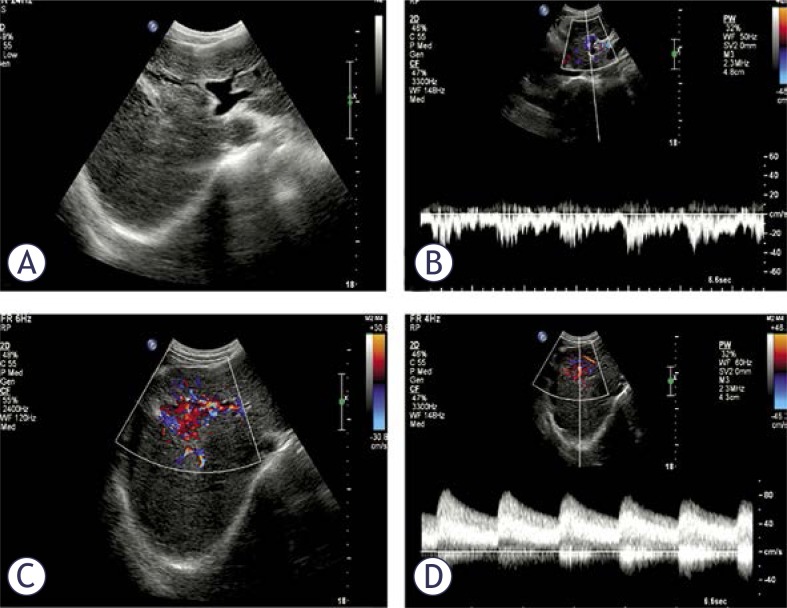
US findings for a typical intrahepatic APF. (A) The dilated draining left portal vein. (B) The arterial-like velocity tracing of the draining portal vein with a continuous hepatofugal flow. (C) The turbulent area of APF. (D) The velocity tracing of a feeding artery with fast flow and a low RI.

**FIGURE 3:**
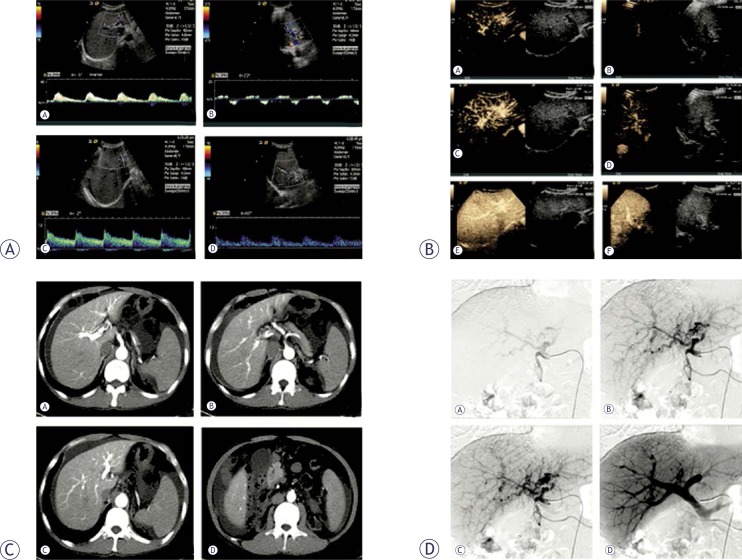
Diffuse intrahepatic APFs with indiscoverable focal lesion, confirmed by multimode imaging findings. (A) Color duplex US images of the intrahepatic branches of the portal vein with normal internal diameter shown as typical arterial-like (a: left branch) or diphase velocity (b: right branch) tracings, as indicated by a systolic hepatofugal dwarf peak and a diastolic hepatopetal low flat shape, and that of the enlarged intrahepatic branches of the hepatic artery, which shows high-velocity flow and low resistivity index (c: left branch, d: right branch). (B) Dynamic CEUS scans show that microbubbles arrived at the affected portal vein and at its parallel running artery in the early arterial phase 7–10 s after SonoVue injection (a: left branch, b: right branch), with the affected portal vein markedly enhanced by the microbubbles during the arterial phase (c: left branch, d: right branch) and which became more echogenic than its surrounding parenchyma until the hepatic veins were stained (e: left branch, f: right branch). (C) The contrast-enhanced CT images show an earlier enhancement of the affected portal vein than that of the superior mesenteric vein during the arterial phase (a: right anterior branch, b: right posterior branch, c: left branch, d: the enhancement of the superior mesenteric artery but no enhancement of its parallel running vein). (D) DSA reveals the opacification of the portal vein following its parallel running artery but no visible fistula during the early arterial phase (a: opacification of hepatic artery, b: opacification of peripheric portal vein, c: opacification of left and right branches of portal vein, d: opacification of superior mesenteric and splenic vein).

An echo-free focal lesion in continuity with a markedly hypertrophied feeding artery and a dilated draining portal vein, which showed a fast and turbulent flow during the CDFI and PW examinations, (Figure 2) was considered a direct indication of APF. The affected portal vein occasionally showed no enlargement with indiscoverable focal lesions. The waveform of its velocity tracing was typically arterial-like or diphase, as indicated by a systolic hepatofugal dwarf peak and a diastolic hepatopetal low flat shape (Figure 3A).

**FIGURE 4:**
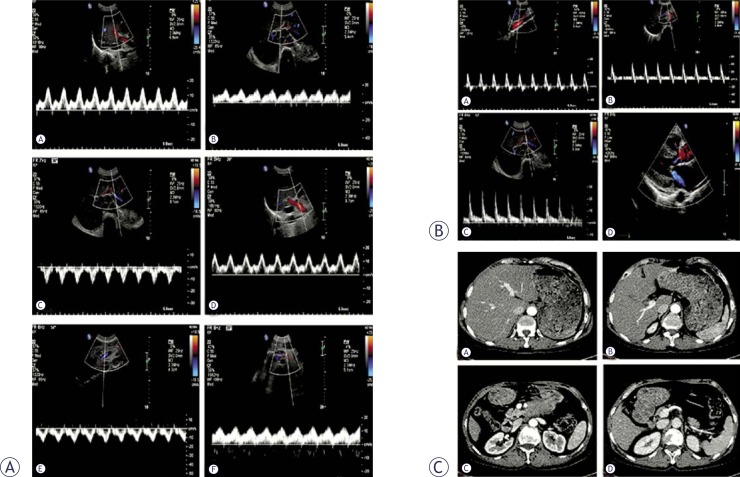
Diffuse extrahepatic APFs with indiscoverable focal lesions, confirmed by multimode imaging findings. (A) Color duplex US images of the portal system with normal internal diameter demonstrate typical arterial-like results with a continuous hepatopetal flow (a: right branch, b: left branch, c: sup. segmental brr., d: main trunk, e: superior mesenteric vein, f: splenic vein). (B) High-resistivity of multiple organs (a: triphase velocity tracing of abdominal aorta, b: triphase velocity tracing of hepatic artery, c: velocity tracing of intrahepatic artery with a high *RI*, d: hypertrophic left ventricular wall with aortic and mitral regurgitation). (C) Stronger enhancement of the affected superior mesenteric and splenic vein than that of the portal vein. (a: left branch, b: right posterior branch c: equal enhancement of superior mesenteric vein and its parallel running artery, d: stronger enhancement of the affected splenic vein than that of portal vein).

The right or left branch of the portal vein draining blood from the intrahepatic APF always had a hepatofugal flow, whereas that in the other lobe had a hepatopetal flow. The flow in the main trunk of the portal vein was always hepatopetal with a markedly decreased velocity in an intrahepatic APF patient but with a markedly increased velocity in an extrahepatic APF patient, whose flow directions in the two main branches of the portal vein were hepatopetal (Figures 2, 3A, 4A).

Dynamic CEUS scans showed that microbubbles arrived at the affected portal vein and at its parallel running artery in the early arterial phase 7–10 s after SonoVue injection: the affected portal vein was markedly enhanced by the microbubbles during the arterial phase and became more echogenic than its surrounding parenchyma until the hepatic veins were stained (Figure 3B).

**FIGURE 5:**
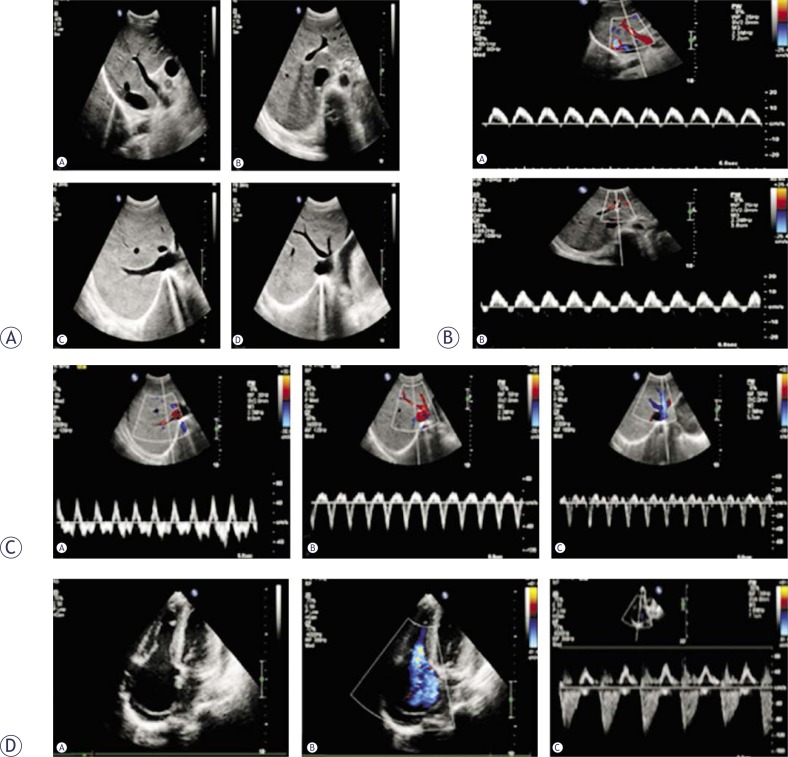
CTS with severe tricuspid regurgitation. (A) Normal US scans demonstrate normal internal diameters of the portal and hepatic veins (a: right branch, b: left branch c: right hepatic vein, d: middle and left hepatic vein). (B) The waveform of velocity tracing in the intrahepatic branch of the portal vein shows a typical hump-like shape with a transitory hepatofugal tracing (a: right branch, b: left branch). (C) PW displays an increase of retrograde phase of hepatic venous flow with increased velocities in the two phases (a: right hepatic vein, b: middle hepatic vein, c: left hepatic vein). (D) Echocardiography shows an enlarged right atrium with severe tricuspid regurgitation (a: Normal ultrasound scans, b: CDFI, c: PW).

Normal US scans and CDFI always showed normal conditions in the portal vein, hepatic veins, and artery. The hepatic veins were sometimes enlarged. The waveform of the velocity tracing in the portal vein, especially its intrahepatic branches, showed a typical hump-like shape with or without a transitory hepatofugal tracing. PW results displayed an increase in the retrograde phase of hepatic venous flow with increased velocities in the two phases. Echocardiography always exhibited an enlarged right atrium with severe tricuspid regurgitation (Figure 5).

### CT findings

The contrast-enhanced CT findings are as follows: (a) earlier enhancement of the affected portal vein compared with the superior mesenteric or splenic vein during the arterial phase or stronger opacification of the affected portal vein compared with that of the superior mesenteric or splenic vein (Figure 3 C); (b) wedge- or irregularly shaped homogeneous enhancement of the liver parenchyma adjacent to the tumor; and (c) earlier enhancement of the affected superior mesenteric or splenic vein compared that of the portal vein during the arterial phase or stronger opacification of the affected superior mesenteric or splenic vein compared with that of the portal vein (Figure 4 C).

### DSA findings

The angiograms demonstrated contrast-filling in the affected portal vein and aneurysmal site of communication between the feeding artery and the draining portal vein during the early arterial phase. Trans-arterial portography revealed a filling defect or nonvisualisation in the affected portal of the intrahepatic APF patient. DSA sometimes revealed early pacification of the portal vein and its parallel running artery, but no visible fistula during the arterial phase (Figure 3 D). 137 suspected CTS based on color Doppler US findings were confirmed by echocardiography, 15 APF by CEUS, 27 APF by CT and 51 APF by DSA. The haemodynamic parameters of *Vmax*, *Vmin*, *Vmax/Vmin*, *PSV*, *RI*, *RV*, *FV*, and *RT/FT* are shown in Table 1. Management and clinical outcomes are shown in Table 2.

## Discussion

The normal liver receives a dual supply of blood from both the portal vein and the hepatic artery. The main portal vein carries venous blood from the intestines and the spleen, while arteries accompany veins and their terminal branches to join via a capillary network. The main portal vein divides into the right and left branches, and the hepatic artery accompanies the portal vein. The terminal branches of the portal vein and their hepatic arterioles are known as the acinus. Blood perfuses the liver parenchyma through the sinusoids and then enters the terminal hepatic venules, which form sequentially larger veins and drain into the inferior vena cava. The portal and hepatic vein are both without valves, thus their haemodynamics, especially the direction of blood flow, are responsive to change when exposed to variations in vascular pressure. The intrahepatic and extrahepatic terminals of the portal vein are both capillary networks; the intrahepatic terminal branches of the portal vein, and the hepatic artery and vein join the sinusoid. The blood flow directions of the portal vein and the hepatic artery are hepatopetal, but that of the hepatic vein is hepatofugal.^16^

Blood, similar to water, follows the path of least resistance. The normal portal venous flow is typically continuously hepatopetal with minimal variations related to the cardiac or respiratory activity.^17^,^18^ If there is communications between an artery and the portal vein, the arterial blood results in an APF and preferentially flows into the adjacent portal venous system with low resistance rather than into the relatively highly resistant arterial lumen. Consequently, the portal drainage is arterialised, so it shows a hepatic artery-like velocity tracing.

APF can be congenital, post-traumatic, iatrogenic (trans-hepatic intervention or biopsy), neo-plastic, or related to ruptured hepatic artery aneurysms.^7^,^19^–^22^ Trauma and neoplasm are the most common causes of APF.^7^ They may be either intra- or extra-hepatic. Intra-hepatic APF can cause the hepatofugal flow of portal drainage in the liver, and extra-hepatic APF can induce the hepatopetal flow.^23^

The portal velocity tracing of CTS is, to a large degree, influenced by the mechanical events in the right atrium.^24^ The main factor is increased hepatic venous outflow resistance with subsequent periodic trans-sinusoidal shunting caused by the elevated right atrial pressure with periodic profuse retrograde venous drainage into the hepatic vein via the inferior vena cava.^25^ This condition is commonly attributed to tricuspid insufficiency, and rarely, constrictive pericarditis.^26^ The pattern is characterized by a monophasic hepatopetal flow with peak velocity and gradual diminution of velocity with or without a transitory hepatofugal flow velocity during each cardiac cycle; it exhibits a periodic hump-like velocity tracing. This pattern is associated with periodic portal hypertension and has been found to be predictive of congestive heart failure.

APF or CTS presentation depends particularly on the shunt flow and, thus, its haemodynamic consequences. A fistula blood flow is a direct function of both the size of the vessel supplying it and the diameter of the communication itself.^13^,^27^ Those with small shunts often present as incidental findings in asymptomatic patients with indiscoverable focuses and may close spontaneously. A large or diffuse fistula results in a decrease in the feeding arterial pressure and an increase in the draining portal vein pressure. A large fistula indicates a varicose or saccular vessel with turbulent blood flow adjacent to the portal drainage.

APF can lead to chronic arterial inflow from the portal vein and CTS can facilitate the hepatic venous reflux into the portal vein. The increased blood flow in the portal system is considered to be the cause of portal hypertension.^7^,^9^,^28^ The increased portal pressure also impairs blood efflux from the spleen and gastrointestinal tract by increasing vascular outflow resistance, which may lead to potential complications.^3^,^4^ Mild to moderate abdominal pain and diarrhea secondary to congestive vascular enteropathy may be some early findings. Signs of more advanced disease include gastro-oesophageal variceal bleeding, splenomegaly, refractory ascites, or other signs of progressive liver failure caused by portal hypertension. APF is one of the main impediments and relative contra-indications to trans-arterial chemo-embolisation, which has been proven to be an effective means of managing unresectable hepatocellular carcinoma (HCC).^19^

The rationale behind AFP treatment is either to overcome existing portal hypertension or to prevent its development. The angiographic embolisation is a simple and effective means of treatment provided that the fistula is not too large and is accessible; it can be performed either via the trans-arterial approach or transvenous approach. The transvenous approach can be considered and used when there are multiple arteriovenous fistulas, or when embolisation via the trans-arterial approach is technically unsuccessful. Closure by embolisation with Gelfoam, steel coils, detachable balloons, n-butyl cyanoacrylate, or bucrylate has proven to be feasible.^2^,^7^,^19^,^22^,^29^ The choice of embolic agent should be based on the underlying mechanism of the shunts and their angio-architecture. The hepatic resection of liver parenchyma containing the AFP and ligation of the feeding artery are reserved for those patients with fistulas that are too large for safe embolization or whose embolised arteries are recanalised.^30^

APF and CTS can be slight and asymptomatic, but they often cause symptoms because of their consequences.^8^,^10^ Both are reversible causes of portal hypertension but utilize different therapeutic procedures. Thus, a correct diagnosis is necessary because only then a definitive therapy can be proposed. The waveform of the velocity tracing of the right and left branches of the portal vein is a reliable indicator that can differentiate the presence of APF or CTS. Its direction can distinguish intrahepatic from extrahepatic APF, and the velocity tracings of the hepatic artery and vein could further support the diagnosis. Therefore, color Doppler US can be proposed for the detecting of APF and CTS. The fistulous mechanism and angio-architecture should be identified in future investigations.

## Conclusions

A fluctuating portal velocity tracing with rhythmicity is a reliable indicator for diagnosing APF or CTS. The evaluation of the velocity tracing patterns of the portal and hepatic veins, especially the direction of portal blood flow, should be used for the routine detection for APF and CTS in patients with gastrointestinal symptoms. Timely diagnosis and application of proper measures can relieve portal hypertension caused by APF or CTS.

## Figures and Tables

**TABLE 1:** The haemodynamic parameters of the portal vein, hepatic artery, and hepatic vein

**Group**	**Portal vein**				**Hepatic artery**		**Hepatic vein**		

	**V_max_ cm/s**	**V_min_ cm/s**	**RI**	**Direction**	**PSV cm/s**	**RI**	**FV_max_ cm/s**	**RV_max_ cm/s**	**RT/FT**
Control	33.88±8.89	25.09±6.52	0.26±0.08	hepatopetal	43.46±4.13	0.72±0.05	34.66±5.83	−14.56±6.37	0.21±0.08
Intrahepatic APF	29.71±7.65	5.17±1.34*	0.81±0.05*	hepatofugal	89.34±10.78*	0.53±0.06*	31.23±6.45	−11.69±5.97	0.24±0.07
Extrahepatic APF	30.03±7.12	4.96±1.23*	0.78±0.04*	hepatopetal	41.53±3.96	0.74±0.04	35.79±6.49	−14.56±6.37	0.223±0.06
CTS	11.03±0.64*	−2.84±0.51*	1.26±0.04*	hepatopetal or with brief reverse	45.13±5.42	0.73±0.03	66.16±14.05*	−35.21±10.45*	1.50±0.23*

Note: *V*_max_, maximum velocity; *V*_min_, minimum velocity; *PSV*, peak systolic velocity; *RI*, resistance index, (*RI* = [*V*_max_ *- V*_min_]/*V*_max_); *RV*_max_, maximum reverse velocity; *FV*_max_, maximum forward velocity; and *RT/FT*, reverse time/forward time.

* P < 0.05 versus control.

**TABLE 2:** Management of APFs and CTSs and clinical outcomes

**Type**		**Cases**	**Etiology**	**Management**	**Outcome(> 6 months)**	
					**Portal velocity tracing**	**Symptom**
Small APF		5	Liver cirrhosis (3) Biopsy(2)	Follow up (2 closures)	No changes except 2 cases (normal)	Asymptomatic
Intrahepatic APF	Focal	33	Liver cirrhosis (6) Hepatoma (22) Congenital(5)	Embolisation of the feeding artery(28) Ligation:too large(5) or recanalised fistulas(4)	Normalization	Relief or asymptomatic (majority)
	Diffuse	7	Liver cirrhosis (1) No (6)	Embolisation of hepatic artery (7) Ligation: recanalised fistulas (1)	Normalization	Relief or asymptomatic (majority)
Extrahepatic APF	Focal	10	Trauma (10)	Embolisation of the feeding artery (8) Ligation: too large(2) or recanalised fistulas(1)	Normalization	Relief or asymptomatic (majority)
	Diffuse	3	Hypertension (3)	Lowering blood pressure (3)	Normalization	Relief or asymptomatic (majority)
CTS		137	Tricuspid regurgitation (137)	Replacement (127) Repair of tricuspid valve (10)	Normalization	Relief or asymptomatic (majority)
